# The Effect of Preimplantation Genetic Screening on Implantation Rate in Women over 35 Years of Age

**DOI:** 10.22074/cellj.2016.3982

**Published:** 2016-04-04

**Authors:** Mina Moayeri, Hojatolah Saeidi, Mohammad Hossein Modarresi, Mehrdad Hashemi

**Affiliations:** 1 Department of Genetics, Tehran Medical Sciences Branch, Islamic Azad University, Tehran, Iran; 2 Omid Infertility Clinic, Tehran, Iran; 3 Department of Genetics, School of Medicine, Tehran University of Medical Sciences, Tehran, Iran

**Keywords:** Preimplantation Genetic Screening, Maternal Age, FISH Technique, Aneuploidies, ICSI

## Abstract

**Objective:**

Advanced maternal age (AMA) is an important factor in decreasing success of assisted reproductive technology by having a negative effect on the success rate of intra-cytoplasmic sperm injection (ICSI), particularly by increasing the rate of embryo aneuploidy. It has been suggested that the transfer of euploid embryos increases the implantation and pregnancy rates, and decreases the abortion rate. Preimplantation genetic screening (PGS) is a method for selection of euploid embryos. Past studies, however, have reported different results on the success of pregnancy after PGS in AMA. Investigating the pregnancy rate of ICSI with and without PGS in female partners over 35 years of age referred to infertility centers in Tehran.

**Materials and Methods:**

In this randomized controlled trial, 150 couples with the female partner over age of 35 were included. Fifty couples underwent PGS and the remaining were used as the control group. PGS was carried out using fluorescent in situ hybridization (FISH) for chromosomes 13, 18, 21, X and Y. Results of embryo transfer following PGS were evaluated and compared with those in the control group.

**Results:**

Implantation rates obtained in the PGS and control groups were 30 and 32% respectively and not significantly different (P>0.05).

**Conclusion:**

PGS for chromosomes 13, 18, 21, X and Y does not increase implantation rate in women over 35 years of age and therefore the regular use of PGS in AMA is not recommended.

## Introduction

As part of the lifestyle in the developed countries, women frequently decide to delay child bearing, which results in an increased incidence of agerelated fertility problems. 

Without accounting for embryo morphology, embryos of women over 35 years of age have shown 40-80% higher rate of aneuploidy (1,3). Due to this increased risk, a low implantation rate and a high abortion rate have been observed after intra-cytoplasmic sperm injection (ICSI) treatment. This observation clearly demonstrates that the age of mother is one of the important factors in predicting a live birth after in vitro fertilization (IVF) (4,6). As a result, although assisted reproductive technology (ART) has been successful, women over 35 years of age still have a very low chance of pregnancy. Therefore, women over the age of 35 form a significant part of those who go under treatment with ICSI. It is thus suggested that screening a healthy embryo for chromosomal abnormalities may improve rate of pregnancy and decrease the possibility of aneuploidy among these individuals. Other factors including hormonal factors, physiological condition of uterus and oocyte quality are effective in the success rate of ART methods, however, chromosomal disorders are directly associated with the age of mother and low rate of ART success (7,9). Evidence shows older women who had failed ART with their own oocyte, became pregnant when they used donated oocytes of younger women (10,11). 

In the IVF/ICSI method, embryo is usually transferred based on embryo development and morphology. However, it is possible that an embryo with a good morphology has a genetic disorder and does not grow. Although preimplantation genetic diagnosis (PGD) was used for couples with a hereditary genetic disorder to diagnose unaffected embryos (12,13), utilizing PGD has enormously evolved and couples who have undergone the IVF treatment opt for this method to select the best embryo. This decision is based on evidence which shows selecting an embryo with normal chromosomes increases the rate of pregnancy and decreases the rate of abortion, especially in older women (14,20). 

Many observations in comparison with the control groups show after preimplantation genetic screening (PGS), the rate of implantation is increased the criterion of research is different based on number of researching chromosomes embryo transfer’s day and number of transferred embryo (21,23). 

In Sweden, the law stipulates the legal transfer of one embryo unless in cases of aged mothers in which two embryos may be transferred. 

A Swedish study, on the role of PGS in aged mothers, reported the low rate of pregnancy in the PGS group compared with the control group and concluded that PGS for aged mothers decreases the rate of successful IVF/ICSI (24). The result of another study in the United States also reported that PGS for aneuploidy not only increases the rate of successful IVF/ICSI, but it can also decrease the rate (25). In contrast to these two studies, the results of three random controlled studies show that PGS significantly improves the rate of pregnancy and implantation. In these studies approximately three embryos were transferred (26). Another study in Italy also reported positive outcome after PGD of aneuploidy in human embryos (19). 

The aim of this study was to investigate the pregnancy rate of ICSI with and without PGS in female partners over 35 years old referred to Tehran infertility centers. 

## Materials and Methods

### Subjects

This randomized controlled trial study was carried out on 150 couples. 163 infertile females over 35 years of age were recruited at a specified time who reffered to Omid Infertility Clinic, Sara Infertility Clinic and the Embryology Laboratory of Aban Hospital in Tehran and this study has been approved by the Vice President for Research of Islamic Azad University, Tehran Medical Branch in accordance with Helsinki declaration and guideline of Iranian Ministry of Health and Medical Education. All the participants signed the consent form and were chosen randomly. Couples with infertility of female or male origin without history of recurrent abortion and with at least two embryos with high morphological quality for biopsy were included. Thirteen infertile individuals who had not undergone a suitable ICSI procedure and as a consequence did not have any embryo for examination, or who had insufficient embryos or their embryos were of low quality for biopsy were excluded from this study. 50 infertile individuals were tested by PGS and the other 100 were considered as the control group. 

### Ovarian stimulation

Primarily, all females were super-ovulated using a gonadotropin-releasing hormone agonist (GnRH) analogue suppression protocol (27) in combination with human menopausal gonadotropin (hMG). Ovulation was induced by a 10000 IU injection of human chorionic gonadotropin (hCG). Subsequently, follicular aspiration was performed 36-38 hours after the hCG was administered (28,29). 

### Embryo culture

The ICSI procedure was used to fertilize the oocytes and performed on mature metaphase II oocytes. Microinjected oocytes were incubated in 25 ml droplets of medium in a Petri dish at 37˚C and 5% CO_2_, 5% O_2_and 90% N_2_. Fertilization was recognized 16-18 hours after injection. Normal fertilization was confirmed by the presence of two distinct pronuclei and polar bodies (3). 

Resulting embryos were cultured for three days. On the third day, tested embryos had a biopsy and underwent PGS examination by fluorescent in situ hybridization (FISH). On the fourth day, as a result of PGS examination, some embryos were reported healthy and among those a maximum of three embryos were transferred. 

### Embryo biopsy

**Fig.1:**
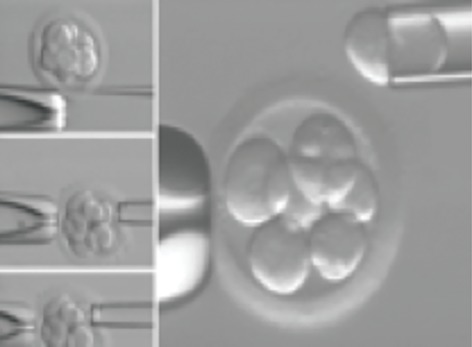
Mechanical biopsy at the cleavage stage of an embryo.

Embryos of grade A, B and less than C with suitable quality were biopsied in third day after fertilization. Embryo biopsy was performed on all embryos that had 5 cells and, 20% fragmentation. Embryo biopsy was performed mechanically by a needle and a biopsy pipette (Fig.1). First, embryos were incubated in Ca²+ free biopsy medium for 15 minutes. Zona pellucida was mechanically drilled with needle and blastomeres were aspirated with a biopsy pipette. In the mechanical biopsy, part of the ruptured zona pellucida overlap after biopsy and heals the damage caused on the embryo. In most cases, one blastomere and in some cases, when there was no suitable nucleus for microscopic analysis, two blastomeres were biopsied. These blastomeres were then genetically tested at Omid Infertility Clinic. 

### Fixation of interphase nucleus

The blastomeres were fixed on a poly-L-lysine cover slide using an acetic acid-methanol solution (1:3). After fixing the nucleus, the location of the nucleus was observed under a phase contrast microscope.

### FISH test steps

Slides were put in 1% formalin (SigmaAldrich, USA) at 4˚C for better stabilization. Next, the slides were washed in 1x phosphate buffered saline (PBS, Sigma-Aldrich, USA) at room temperature for 5 minutes and were then placed in pepsin (Merck, USA) at 37˚C for 5 minutes for better cytoplasm digestion (pepsin solution contains 0.05 g pepsin in 100 ml HCL 0.01 N). The slides were rewashed for 5 minutes with 1x PBS buffer at room temperature. Subsequently, three ethanol solutions (SigmaAldrich, USA) (70, 85 and 100%) were used for 1 minute for better wash. Finally the slides were immersed in methanol for 5 minutes to be dehydrated. 

Five color Vysis probe kit (Multi-Vysion™ PGT multi-color Probe Panel, Vysis Inc, USA) designated for chromosomes X, Y, 13, 18 and 21 marked with green, aqua, red, blue and gold colors was used to recognize 5 chromosomes at the same time. 

Probes were poured on the cell nucleus and were covered by a cover slip and then fixed by paste. The cover slides were then placed at 76˚C heat for 5 minutes to denature the DNA and the probe elements. Heated slides at 37˚C were put into a moist chamber for 4 to 8 hours; lesser time made the signal weak and the more time increased the background color with both making the signal reorganization difficult. After, slides were washed in 0.4X saline-sodium citrate (SSC) buffer (Sigma-Aldrich, USA) at 73˚C for 5 minutes. The slides were then placed in 2X SSC solution for 1 minute and placed in distilled water for 1 minute. 

Finally, slides were dried in room temperature and signals were observed under a fluorescent microscope by using Antifade II. 

### Observable scales in FISH examination

**Fig.2:**
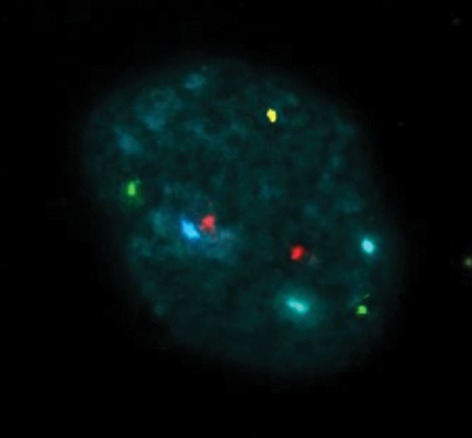
Normal male embryo after PGS. Photo was taken by applied spectral imaging software. A blastomere nucleus hybridized with probes specific for chromosomes 13 (red), 18 (aqua), 21 (green) X (blue) and Y (gold). The signal pattern is consistent with normal male chromosome complement. PGS; Preimplantation genetic screening.

In size and light degree, they are considered two signals, when the signals were too weak or totally unrecognizable, it was reported as no signal, and when all five chromosomes analyzed were present in the correct numbers, the embryo was reported as normal (Fig.2) and when there were unequal numbers of chromosomes, abnormal embryo or aneuploidy was reported.

### Embryo transfer

Generally, two normal embryos with the best morphologic qualities were selected for transfer on day four. Embryos were ranked according to their morphologic qualities with a focus on the regularity and number of blastomeres, and the percentage of fragmentation (29). In the control group, the selection of embryos for transfer was based only on morphologic qualities according to the scoring procedure described above. A maximum of three embryos were transferred four days after injection in both study groups although two embryos were usually transferred. 

### Outcome measures

Biochemical pregnancy and implantation was defined when serum β-hCG levels reach above 10 IU per liter after 3 weeks of embryo transfer. Clinical pregnancy was defined as the presence of a gestational sac confirmed by transvaginal ultrasound examination at a gestational age of 7 weeks. In this study, we evaluated implantation rate by measuring the level of β-hCG. 

### Statistical analysis

We used Fisher’s exact test for statistical analysis and generated 95% confidence intervals (CIs). P>0.05 was reported as not significant. 

## Results

**Fig.3:**
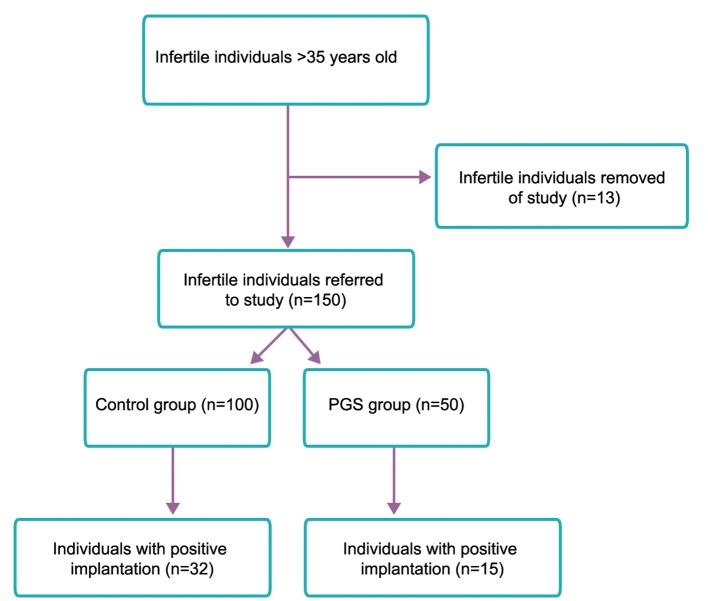
Patient flow diagram. PGS; Preimplantation genetic screening.

Totally 50 infertile individuals were tested of which five, after PGS, did not obtain healthy embryos for transfer (non-transfer rate of 10%). Thirty individuals, following transfer of healthy embryo, did not become pregnant (implantation failure rate of 60%) and the other 15 individuals, after transfer, did not show a positive β-hCG test for biochemical pregnancy (implantation rate of 30%) (Fig.3). 

Of 50 infertile individuals who were tested, 324 embryos were biopsied and tested by FISH. A total of 279 embryos generated signals and no signal was observed in 45 embryos. From 279 tested embryos, 125 embryos (44.8%) were reported as abnormal and 154 embryos (55.2%) were reported healthy for the aneuploidy of chromosomes X, Y, 13, 18 and 21 (Table 1). 

**Fig.4:**
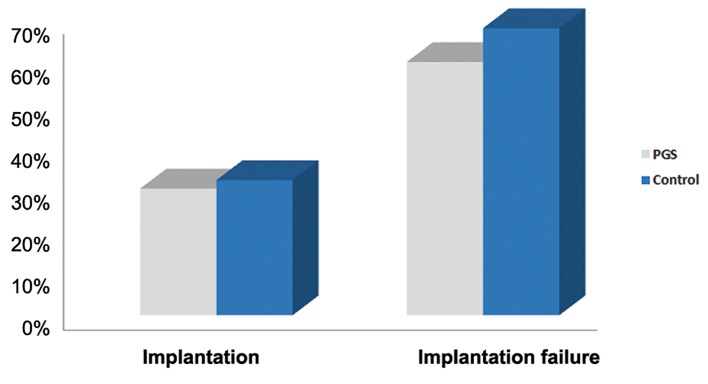
Comparison of implantation rates with and without the use of PGS in women aged above 35. PGS; Preimplantation genetic screening.

In the control group from 100 mothers aged above 35, 32 mothers were pregnant (implantation rate of 32%) and 68 mothers were not biochemically pregnant (implantation failure rate of 68%) (Fig.4). The difference of implantation rate between cases and controls was 2%. This difference between the two groups was not significant, suggesting PGS had no effects on the implantation rate in mothers aged above 35. 

**Table 1:** PGS cycle data


FISHresults	n	%

Biopsied(day3,cleavagestage)	324	100
Nosignal/inconclusive	45	13.88
Normal	154	47.53
Abnormal	125	38.58
Aneuploidy chromosome 21	56	44.80
Aneuploidy chromosome 18	45	36.00
Aneuploidy chromosome 13	35	28.00
Aneuploidy sex chromosome	70	56.00


PGS; Preimplantation genetic screening and FISH; Fluorescent in situ hybridization.

## Discussion

In this study, we have shown that preimplantation genetic screening did not increase. 

There are two randomized controlled trial which were published and did not represent any advantage for using PGS as an indicator of advanced maternal age (AMA) (3,26). In Staessen et al. (3), 400 women aged above 37 were randomly selected. The first result showed that there were no statistical difference in implantation rate, the rate of pregnancy per transfer and the rate of pregnancy per cycle between the PGS and the control groups. In a similar study by Stevens et al. (30), 40 women aged above 35 were randomly selected. There was no signification difference in clinical pregnancy or the rate of implantation of the PGS group compared with the controls. 

It was hypothesized that selecting an embryo which has normal chromosome numbers will increase the rate of implantation and pregnancy and will decrease the rate of abortion, but both studies showed that PGS had no effect. Nevertheless, such a result cannot be exactly determined. There are many factors which affect this achievement of which one is embryo biopsy. Many studies have shown that the biopsy from embryo has no effect on the growth of embryo in laboratory conditions but the result of pregnancy rate indicated that it is still necessary to review this case (24). It is possible that blastomere biopsy on day three of embryonic stage prevents the potential of an embryo to successfully implant (31). However, the effect of biopsy alone on pregnancy rate has not been studied. 

Moreover, the omission of one or two cells may have a detrimental effect, and this may prevent the growth of the embryo. On the other hand, the selection of an embryo with 5 normal chromosome sets (13, 18, 21, X and Y) can not guarantee the absolute growth and health of the embryo. FISH usually estimates between 5-8, rather than all 23, chromosome pairs (32). Recent studies have shown that embryonic aneuploidy occurs in clinically significant amounts for all 23 chromosome pairs (33). FISH is thus unable of diagnosing many of the chromosomal abnormalities generally found in the embryonic developing stage. Therefore, the limitation in the number of chromosomes that can be analyzed with FISH could lead to the transfer of normal embryos that are in fact aneuploid for one or several chromosomes not tested. This problem may be overcome in the future by the use of new techniques such as array comparative genomic hybridization (aCGH) in which the complete ploidy for a blastomere can be detected after biopsy (34). 

PGS on the biopsied blastomeres from developing embryos presents its own challenges. Cell mosaicism is also a main factor in generating false results in PGS. A normal embryo may be incorrectly reported abnormal because of mosaicism, and may not be transferred, or vice versa. In contrast an abnormal embryo may be reported as normal and may be transferred but it will not grow and pregnancy will fail (24). In addition, many human embryos generated by ICSI may be mosaic (3,35,36) where chromosomal constitution of the one blastomere may not be representative of the entire embryo. Studies have repeatedly demonstrated that, at day three of development, embryos have high levels of mosaicism (37,38). Mosaicism is a situation in which a single embryo is made of more than one distinct genetic cell line. In other words, mosaic embryos may have both euploid (normal) and aneuploid (abnormal) cell lines inside them. Studies estimating this event have deduced that the majority of all embryos may be mosaic at day three of development (37,39). Therfore, a biopsy performed at day three of development may produce a result that is not representative of the overall embryo. Also, although mosaicism has been shown to exist at day five of embryo development (40), recent studies suggest that mosaicism may be much decreased by day five of development (28,41). 

When we extract a normal cell from an abnormal embryo, this decreases the ratio of normal cells in the embryo and thus prevents the growth and progress of that embryo. On the contrary, when we extract an abnormal cell out of a normal embryo, the embryo grows better while this embryo was not transferred for the wrong diagnosis (24). 

Another important factor is the day of biopsy. Blastocysts are more likely to be the appropriate source for biopsy, because more cells can then be produced and analyzed with accurate clinical results (42). However, usually for performing PGS, the most popular sources are either polar body biopsy (15,16) from the oocyte or cleavage stage biopsy on one or two blastomeres (12). 

Transfer of embryos in the two study groups was made on distinct days. Transfer was performed on day four because we wanted to keep each step for the control group the same as our ordinary routine as much as possible. 

## Conclusion

We show that PGS, which is used to select embryos for transfer, does not improve the rate of implantation for advanced maternal age. Also the results showed that there was no significant difference in implantation rate for transfer between the PGS and control groups. 

Our study was limited to women undergoing PGS as an indicator of advanced maternal age. It remains uncertain whether the results would be different for women with other indications for PGS. 

The hypothesis that PGS in women of advanced age improves the implantation rate has not been supported by this study. Therefore, this study and previous randomized controlled trials alike show that the regular use of PGS in AMA is not recommended. 
